# British Health Stations.—I

**Published:** 1904-02-13

**Authors:** 


### BRITISH HEALTH STATIONS.—I.

(By Our Special Medical Commissioner.)

Travel has for long been recognised as an attractive pursuit, rich in educational advantages; and while residence in so-called health resorts has been favoured by fashion, it has also been found to be of the greatest benefit in meeting actual physical needs. But with the coming of fresh light on the problems of disease, and the establishment of reliable knowledge respecting the causation of morbid processes, increased attention has been given to a serious study of the important factors which go to the making of a satisfactory health resort. Modern views of pathology have gone far to recast our conceptions of disease and modify much in the methods of treatment. We are learning that hygienic measures long recognised as necessary for the maintenance of vigorous health, and desirable for the prevention of disease are also essential to the speedy and effectual restoration of the sick. Much of the empiricism and superstition which have for so long clung tenaciously to medicine as survivals of mediæval days, and the period of primitive prophylactic effort, are being ruthlessly swept aside and both the theory and art of medicine are being rapidly reconstructed on a sound scientific foundation, which is already affording a sure basis for an upbuilding on such rational lines as were never dreamt of by the ancient dealers in physic.

From earliest days certain districts have been famed for their virtues in healing and alleviating the afflicted, but usually a resort to such was only undertaken under the direction of superstition, tradition, or under the guidance of influences altogether alien to the scientific spirit of to-day. Now, however, a more or less rational procedure may be followed in the selection of cases for places, and places for cases. The haphazard method is being discarded. Reliable data are forthcoming regarding climatic and other conditions existing at the various health stations; and much trustworthy information is now available respecting the influence of such agencies on different physiological and pathological states. The psychological aspect of health resorts may also be well considered by medical men. Wisely conducted travel or residence in a suitably selected climate often prove of the greatest prophylactic service, and in the arrest or relief of many pathological processes resort may advantageously be had to various climatological or balneological stations in this country.

It is unnecessary here to discuss the different features comprised in what we term climate; and any attempt to indicate in anything like fulness the way in which morbid conditions may be modified by environment or influences associated with or dependent on special localities would take us too far afield. For practical purposes, however, it is most desirable that every medical practitioner should at least be well acquainted with the claims of the various health stations of his own country, and know sufficient regarding their advantages and disadvantages to direct patients with various ailments and of different constitutions and dispositions in the choice of a desirable resort.

We purpose, therefore, in a series of short articles, to deal in a thoroughly practical fashion with the more important of our British health stations, limiting our view mainly to that of the busy general practitioner who would secure the maximum of useful information in the minimum of time and with an irreducible expenditure of energy.

As far as possible the articles will be based on personal investigation or special research, and every care will be taken to obtain the most reliable information available.

We trust these short papers will not only be of benefit to patients and service to practitioners, but will do something to stimulate local enterprise at the various health stations in this country.

